# A Report of Guillain–Barré Syndrome With Myalgia and Mild Weakness

**Published:** 2014

**Authors:** Vahid AMINZADEH, Afagh HASSANZADEH RAD

**Affiliations:** 1 1.Pediatrics growth disorders research center, 17 Shahrivar Hospital, School of Medicine, Guilan University of Medical Sciences, Rasht, Iran

**Keywords:** Guillain-Barré syndrome, Myalgia, Weakness, Children

## Abstract

We report a rare case that revealed severe myalgia as the chief complaint that is not mentioned in the list of frequent symptoms of Guillain Barré.

Guillain-Barré syndrome (GBS) is an acute inflammatory demyelinating polyneuropathy (AIDP).Required features for diagnosis of GBS are progressive motor weakness of more than one limb and areflexia. We report an 11-yearold boy who was referred to the emergency department with complaints of generalized body pain and gate problem.

It seems that if myalgias are the chief complaint and weakness is mentioned as a less important symptom, clinicians should consider GBS after ruling out other reasons for myalgia especially inflammatory myositis.

## Introduction

Guillain-Barré syndrome (GBS) is an acute inflammatory demyelinating polyneuropathy (AIDP), an autoimmune disease affecting the peripheral nervous system that causes ascending paralysis and the loss of deep tendon reflexes (1).

It is a heterogeneous group of disorders with similar clinical presentations. Typically, it is an acute, self-limited illness, which peaks in 2 to 4 weeks and then subsides (2). The pathogenesis of GBS remains unclear. Increasing data indicate that it is an autoimmune disease, often triggered by a preceding viral or bacterial infection with organisms such as Campylobacter jejuni, cytomegalovirus, Epstein-Barr virus, or Mycoplasma pneumoniae. Vaccination against the flu, rabies, and meningitis are also documented precipitating factors that have been reported (3).

Since poliomyelitis has nearly been eliminated, the Guillain–Barré syndrome is currently the most frequent cause of acute childhood flaccid paralysis worldwide and constitutes one of the serious emergencies in neurology (4).

Men are1.5 times more likely to be affected than women, and the incidence increases with age from1 per 100,000 in those below30 years of age to about4 cases per100,000 in those older than 75 years of age (5).

The natural history of GBS in infants and children is more variable and more benign than in adults. Although, affected children usually recover in a shorter time than adults do. However, the mortality rate has been reported as3-5% (6).

Required features for diagnosis of GBS are progressive motor weakness of more than one limb and areflexia. In addition, the existence of the progression of weakness, relative symmetry, mild sensory symptoms, or signs and cranial involvement could strongly support diagnosis (7).

Meanwhile, the main feature is progressive bilateral and relatively symmetric weakness of the limbs that progresses over a period of12 hours to28 days before a plateau is reached (8).

However, in this report, investigators report a rare case that revealed severe Myalgia as the chief complaint that is not mentioned in thelist of frequent symptoms of GuillainBarré.

## Case report

An 11-year-old boy was referred to the emergency department with complaints of generalized body pain and gate problems. He revealed a history of URI in the preceding 2weeks and mentioned generalized body pain3 days prior to admission and gate problems from the day before. No past medical history or family history were mentioned.

In a systemic examination, his vital signs were stable and generalized muscle tenderness existed.

Other systemic examinations were normal. 

A neurological examination showed normal mental status, cranial nerve, and sensory examination.

The deep tendon reflex (DTR) in upper limbs and lower limbs were diminished (+1) and the proximal and distal muscle force in the upper and lower limbs were4/5. He could not walk due to severe pain. 

The patient mentioned pain as chief complaint, which is rare. The clinicians performed two electromyography (EMG) and nerve conduction velocity (NCV) tests to ensure the diagnosis. Our laboratory results are as follows: 

WBC: 9000

Lymphocyte: 60%

PMN: 40%

Hb: 11

CRP: +1

RF:negative

Wright Test: negative

ESR: 16

CK: 75

Repeated CK: 90

LDH: 290

Repeated LDH: 300

CSF      exam:WBC:0,      RBC:0,Protein:55,Glucose:80 (concomitant blood sugar:100)

Lumbosacral MRI: normal

Technetium bone scan: normal

EMG NCV: Diagnostic for Guillain-Barré (Demyelinating polyneuropathy)

Repeated EMG NCV: Diagnostic for Guillain-Barré (Demyelinating polyneuropathy)

From the laboratory results and positive (EMGNCV) tests, Guillain Barré was diagnosed and he was treated with standard drugs and released after10days. In a follow up, he mentioned no weakness, he could walk, and his pain was decreased.

## Discussion

As GBS is a heterogeneous group of disorders with similar clinical presentations and has challenges regarding itsdiagnosis (2), a thorough medical assessment may be needed to aid clinicians to differentiate symptoms and signs of GBS from other diagnoses.

According to the literature, required features for diagnosis of GBS are progressive motor weakness of more than one limb, areflexia, the existence of progression of weakness, relative symmetry, mild sensory symptoms or signs, and cranial involvement could strongly support its presence7.

These features were similar with the results mentioned by Ismael et al that revealed clinical manifestations of28 children diagnosed as GBS were motor weakness(100%),cranial nerves involvement(14.29%), and disturbance of sensation(17.80%) (6). 

In addition, in a series of49 children hospitalized with GBS, not surprisingly, weakness was the most common initial complaint and, then, pain (particularly in the back and lower extremities) was a prominent feature (7).

Furthermore, Hicks et al identified 35 pediatric patients with Guillain-Barré that showed that the most common presentation of symptoms were paresthesis(54%), weakness(49%), and Myalgias (49%). Sensation was affected in54% of patients and hyporeflexia or areflexia were present in 94% of patients. Cranial nerve dysfunction (46%).also, and autonomic involvement e.g., changes in blood pressure, pulse, or bowel/bladder control) were also common (9).

Borade et al. indicated a case that presented a GBS 2-year-old male child admitted to hospital with a history of acute onset of bilateral lower limb progressive leg weakness and difficulty in walking and standing. In this study, no complaint of pain was mentioned (10). 

Although the disease is characterized by a rapidly ascending weakness, some variants present with atypical clinical features1.

Roodbol et al conducted a study with 23 preschool children and in32 older children with GBS. Their results mentioned that for preschool children, refusal to walk and pain in the legs was the most frequent presenting symptom (65%),while older children presented with more classic symptoms of weakness and paresthesias (11).

Although,in some of these investigations pain was indicated as a GBS feature. It should be mentioned that in these cases, patients who encountered pain that was accompanied by other frequent symptoms of GBS and Guillain-Barré syndrome pain involvement had not been considered as a major clinical manifestation for diagnostic criteria among children, which differs with our study. Our study shows no other symptoms except pain.

Myalgia had not been considered a chief and common complaint of GBS. Often patients report weakness with or without myalgia. However, results showed that if myalgias were the chief complaint and weakness was mentioned as a lessimportant symptom, clinicians should consider GBS after ruling out other reasonsfor myalgia especially inflammatory myositis. Hence, a thorough assessment of effective factors that can treat weakness and gate disability sooner and preserve patient life better is of clinical significance.
